# Generation of conditional knockout alleles for PRL-3^☆^

**DOI:** 10.1016/S1674-8301(11)60058-4

**Published:** 2011-11

**Authors:** Hong Yan, Dong Kong, Xiaomei Ge, Xiang Gao, Xiao Han

**Affiliations:** a Key Laboratory of Human Functional Genomics of Jiangsu Province, Nanjing Medical University, Nanjing, Jiangsu 210029, China;; b Model Animal Research Center, Nanjing University, Nanjing, Jiangsu 210093, China.

**Keywords:** PRL-3, conditional knockout alleles, Cre recombinase, tumor metastasis

## Abstract

Phosphatase of regenerating liver-3 (PRL-3) is a member of the protein tyrosine phosphatase (PTP) superfamily and is highly expressed in cancer metastases. For better understanding of the role of PRL-3 in tumor metastasis, we applied a rapid and efficient method for generating *PRL-3* floxed mice and investigated its phenotypes. A BAC retrieval strategy was applied to construct the *PRL-3* conditional gene-targeting vector. Exon 4 was selected for deletion to generate a nonfunctional prematurely terminated short peptide as it will cause a frame-shift mutation. Conditional knockout *PRL-3* mice were generated by using the *Cre-loxP* system and were validated by Southern blot and RT-PCR analysis. Further analysis revealed the phenotype characteristics of PRL-3 knockout mice and wildtype mice. In this study, we successfully constructed the *PRL-3* conditional knockout mice, which will be helpful to clarify the roles of PRL-3 and the mechanisms in tumor metastasis.

## INTRODUCTION

The majority of cancer related deaths are induced by tumor metastasis and recent oncologic researches have focused on the mechanisms of metastasis. Protein tyrosine phosphatases (PTPs) play a fundamental role in regulating diverse cellular processes, including cell proliferation, adhesion and migration[1]. Phosphatase of regenerating liver-3 (PRL-3) is a member of the protein tyrosine phosphatase (PTP) super-family. Mouse PRL-2 and PRL-3 exhibit 87% and 76% homology to mouse PRL-1 in their amino acid sequences, and three mouse PRL proteins contain a C-terminal consensus sequence for prenylation[2]. *PRL-3* encodes a small, -20 kDa tyrosine phosphatase that is located at the cytoplasmic membrane when prenylated at its C-terminus; however, the unprenylated *PRL-3* is shifted into the nucleus[3]. The mouse *PRL-3* gene has 6 exons, encompassing 30.36 kb of genomic DNA.

In human diseases, PRL-3 is highly expressed in cancer metastases. Moreover, overexpression of PRL-3 also facilitates the cells to form metastasis-like tumors in mouse models. High expression of PRL-3 has been found to be associated with human colorectal tumors[4]–[6]. We have reported that PRL-3 promoted motility and metastasis of mouse melanoma cells[7]. Recently, it has also been reported that silencing of the *PRL-3* gene by siRNA reduced cell migration in the multiple myeloma cell line INA-6[8]. PRL-3 shows promise as a biomarker and prognostic indicator in colorectal, breast, lung and gastric cancers[9]–[12]. However, the substrates and molecular mechanisms of action of the PRLs have remained elusive. Recent findings indicate that PRL-3 may down-regulate PTEN expression and signal through PI3K to promote epithelial-mesenchymal transition[13]. In conclusion, PRL-3 is specifically expressed in malignant plasma cells and may have a role in regulating migration of these cells. The progression of other tumor types was positively correlated with PRL-3 expression[14], suggesting that PRL-3 is a molecular marker for metastatic tumor cells and could be an important target for anticancer therapeutics[5],[15],[16]. However, the substrates and molecular mechanism by which PRL-3 promotes tumor metastasis are far from clear. To further reveal the physiological significance of *PRL-3*, we created the *PRL-3* conditional knockout mice through the *Cre-loxP* system.

## MATERIALS AND METHODS

### Construction of *PRL-3* conditional gene-target vector

The *PRL-3* conditional gene-targeting vector was made based on a BAC-retrieval strategy as described previously[17]. Briefly, the region of the BAC containing the *loxP* sites and positive and negative selection markers is then excised from the BAC and transformed into ES cells (BAC from Sanger, 129S7/AB2 clone). After validation by DNA sequencing, the BAC was electroporated into EL350[18]. Retrieval primers with Amp and TK using pL253 (pBluescript-based vector) as template and in which the restriction sites for *Not*I and *Ava*I were removed. Restriction sites for these enzymes were included in the amplification primers to permit directional cloning of the PCR products into PL253. The 5′ homolog (5′-CTACCTCCTACACCTGAGAACAGCTCAGCACTCCACTCCAAGTCAGTGCCATGGCTCACAGCTAGTCCCTGCCACCATTC-gcggcc-GCACGAATGGGTTACATCGAACTGGATC-3′) and 3′ homolog (5′-CTGCCCAGGTCTTGATGTGGTGGTGACGGCTGTGTAGGCATTTGATGGCCTGTGATAGGTGGATGCTGAACTCCTCCTGG--TAGAACTAGTGGATCCTCTAGAGTCG-3′) were designed for retrieving. The next step was introduction of a loxP site into the subcloned DNA. PCR amplified 80-bp homologous arms loxP-NEO-loxP cassette from PL452. This is accomplished by introducing a floxed neomycin resistance (Neo) cassette (PL452) *via* homologous recombination into the subcloned plasmid DNA, and by removing the DNA fragment between two loxp sites *via* Cre recombinase induced by 0.1% arabinose. Primers designed for inserting the first loxP between exon 4 and exon 5 were 1Lox-F (5′-GCAGGGAGGGCTTCCTGCTCTGATCCAGTCACTGCACCTCATGCAGAAAAAGAGCTGCCGCAAAGACATCCTGGTCATCCAGCAGCCCAATTCCGATCATATTCAATAACC -3′) and 1Lox-R (5′-CCTCCTGCAATAGGGGTGTCGGCAGGCGCTCTCCTGCCTGGCTCACTTCCATCTAGTGTGAGCAGCTGTGACTCAAGCAAGGGTCAAGCGCTCTAGAACTAGTGGATCCCCTCGAGGGACC-3′). Then, Prl-3 first *lox*P popped-out plasmid was transformed into EL250 with the final step the introduction of a second loxP site into the subcloned DNA. PCR amplified 80-bp homologous arm FRT-NEO-FRT-loxP cassette from PL451[17]. Primers 2Lox-F (5′-AGCCTCTCATCGCCTGCCATGTGGGAGCCTGTCTCCTGGCAGCATTGCATCCCTAGAGACCCTGTGCAGAGGTCGTGGGTAG_CGACGGTATCGATAAGCTTGATATCGAATTCC-3′) and 2Lox-R (5′-TCTGCTGAGCGAGAAGCCTGGCTTATCCAGATTCTGCACTGGGAAGAGAGGCCTTGTTCCAGGAAACTAACAGGCCAGGCTAGAGGT_GCTCTAGAACTAGTGGATCCACCTAATAACTTC-3′) were designed. Second PRL-3 loxP plasmids were transformed into DH-5α to get the purified targeting vector.

### Generation and genotyping of conditional *PRL-3* knockout mice

The gene-targeting vector was linearized with *Not*I and then electroporated into W4 ES cells. The transfected ES cells were selected with Dulbecco's Modified Eagle Media containing G418 (250 µg/mL) and gancyclovir (2 µmol/L). The double G418 and gancyclovir resistant ES clones were screened by PCR (forward: 5′-GAATCTTACAACCCAGCCCCAAC-3′, and reverse: 5′-ATGAGCATACTGAATGAGTCCACAC-3′). Southern blot analysis for the cor-rect targeted allele using *Eco*RI digestion and the 3′external probe (SouthP3-forward: 5′-GTGGAAGACAGGGAGATACC-3′ and SouthP3-reverse: 5′-CAAGCTAGGAAACACTAGAACC-3′) and 5′ external probe (SouthP5-forward: 5′-CCTTGTTACTTCCACACCAG-3′ and SouthP5-reverse: 5′-TGAGAAACCCTGTATTACGTG-3′) were designed for Southern blots by detection of the *Eco*RI sites. The targeted ES clones were injected into blastocysts from c57BL/6J mice.

Chimeric mice were bred with CAGGS-Flpe (The Jackson Laboratory) transgenic mice to delete the FRT flanked Neo cassette to produce the PRL-3 flox allele, which was then bred with EIIa-Cre line to delete the floxed exon 4 to generate the PRL-3⊿exon4 mice. All these alleles were maintained in the c57BL/6J background.

Genotyping was determined by PCR analyses of genomic DNA isolated from mouse tails by proteinase K digestion and phenol/chloroform extraction. To genotype the PRL-3 flox allele, primers PRL-3-1F (5′-CAAGGTCTTTCTTTCACAGCCTGG -3′) and PRL-3-1R (5′-TGAGCATACTGAATGAGTCCACAC-3′) were designed to detect the loxP-FRT sites that remained after removal of the Neo cassette by Flpe-mediated recombination (***Fig. 3A***). To detect the deletion of the floxed exon 4 from the PRL-3 flox allele (***Fig. 3B***), primers PRL-3-1 F (5′-CACTCCCACTGTCCTTTCCT-3′) and PRL-3-1R (5′-GCCTGGCTCACTTCCATCTA-3′) were applied.

### RT-PCR and Northern blot analysis

Total RNA of the heart, brain and muscle from wild type and Prl-3 homozygous mice were extracted using TRIzol (TaKaRa, Dalian, China), and 1 µg RNA was used for reverse transcription (RT) using PrimeScript (TaKaRa, Dalian, China). One µl RT was applied for PCR using a forward primer located in exon 2 and a reverse primer located in exon 6 (***Fig. 3C***). RT-PCR primer sequences were RT-forward (5′-TTCCTCATCACCCACAACC-3′) and RT-reverse (5′-AACCTCAGTCTCTGCTTAG-3′). Total RNA was prepared from the heart, muscle and brain using the Trizol reagent (Life Technologies, GIBCO-BRL, USA). Twenty µg RNA was subjected to electrophoresis in a 1.0% (w/v) agarose gel containing formalin. After transfer, the nylon membrane (Hybond N, Amersham Pharmacia) was hybridized with ^32^P-labeled probes for mouse *PRL-3*. cDNA PCR fragments were used as probes for *PRL-3*, which were *PRL-3* CDS182 (5′-ACGGCATCACTGTTGTG-3′) and PRL-3 CDS349 (5′-GAGCCACGAGCACTGG-3′).

### General characteristics of *PRL-3* knockout mice

We measured the body weights of *PRL-3* homozygous (*PRL-3*^−/−^) mice and wildtype (*PRL-3*^+/+^) mice continuously for 9 weeks and established the growth curve of body weights of *PRL-3* knockout mice. Each group had 9 mice and their body weights were measured in g at 1, 2, 3, 4, 5, 6, 7, 8, and 9 weeks. For further phenotype analysis, blood chemistry indicators were measured of the plasma of *PRL-3*^−/−^ mice and *PRL-3*^+/+^ mice. Each group has 8 mice, which had been fasted for 16 h before sacrifice. Blood collected from mice were put into Eppendorf tubes for aqueous bath at 37°C for 30 min. Then, blood samples were centrifugated at 3,000 rpm for 10 min and the plasma was separated and collected. We detected the plasma levels of cholesterol, triglycereide, high density lipoproteins, low density lipoproteins (LDL) and glucose in *PRL-3*^−/−^ and *PRL-3*^+/+^ mice.

Intraperitoneal glucose tolerance test (IPGTT) was then performed in six *PRL-3*^−/−^ mice and six *PRL-3*^+/+^ mice were selected at 12 weeks of age. Glucose tolerance measurement was conducted at the second morning after fasting 16 h overnight. Mice were intraperitoneally injected with D-α glucose (0.15 g/mL, dilute in MillQ) at a dose of 1.5 mg/kg. Finally, the values of blood glucose were measured at 0, 15, 30, 45 and 60 min by using Blood Glucose Test Strips.

### Statistical analysis

Data were analyzed with Student's *t*-test and presented as mean±SD. *P* < 0.05 was considered to have significant difference.

## RESULTS

**Fig. 1:**
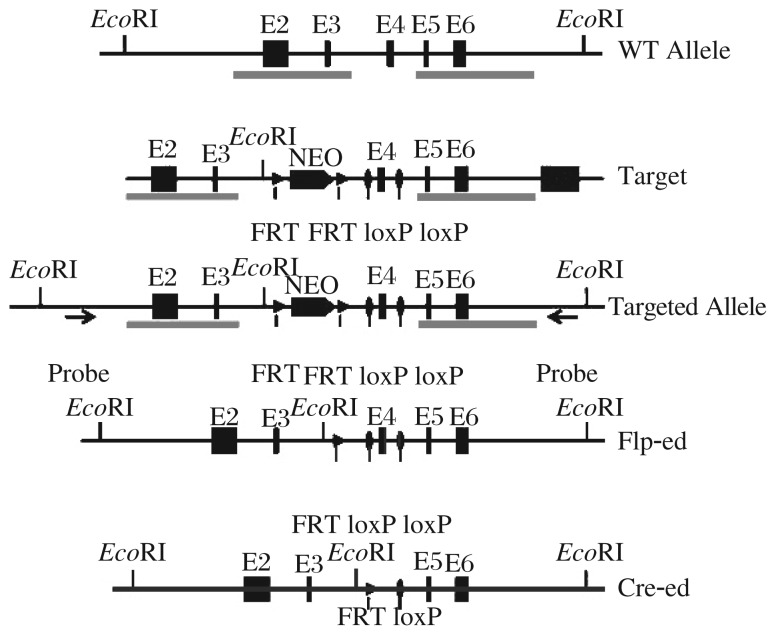
Gene-targeting strategy for constructing the *PRL-3* conditional knockout alleles. A schematic representation of the *PRL-3* conditional gene-targeting vector based on a BAC retrieval strategy. The *PRL-3* gene-targeting vector contains 5′ and 3′ homology arms, FRT flanked Neo cassette, floxed exon 4 and the HSV-TK negative selection marker.

To establish the conditional knockout alleles through Cre-mediated excision, we decided to target exon 4 of the *PRL-3* gene by flanking it with two loxP sites. Deletion of exon 4 is predicted to generate a nonfunctional prematurely terminated short peptide as it will cause a frame-shift mutation. We applied a BAC retrieval strategy as described previously[17] to construct the *PRL-3* conditional gene-targeting vector, which contained 5′-and 3′-homology arms, FRT flanked Neo cassette, floxed exon 4 and the HSV-TK negative selection marker (***Fig. 1***).

For gene targeting, 20 µg of *Not*I linearized targeting vector[19] DNA was electroporated into 1×10^7^ W4 ES cells. Positive clones were obtained after G418 selection and screened by long-range PCR. The PCR primers are depicted in ***Fig. 1***. Two clones had undergone correct homologous recombination on the 3′ site and 5′ site and that the two loxP sites were properly inserted.

**Fig. 2:**
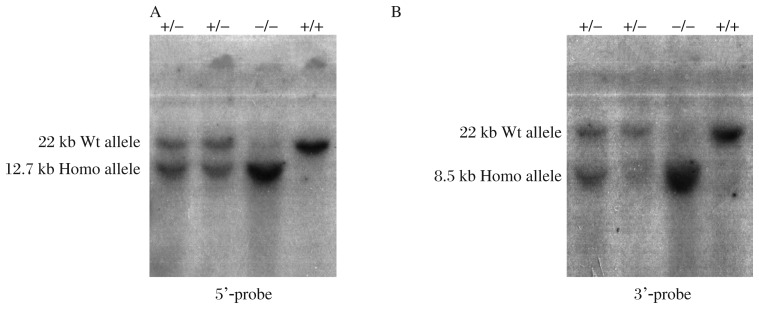
Southern blots analysis. Targeted clones were validated by Southern blot with 5′ (A) and 3′ (B) probes. The results differentiated the *PRL-3* flox mutated allele (8.5 kb, 12.7 kb) from the wildtype (wt) allele (22 kb). Homo: homozygous.

**Fig. 3:**
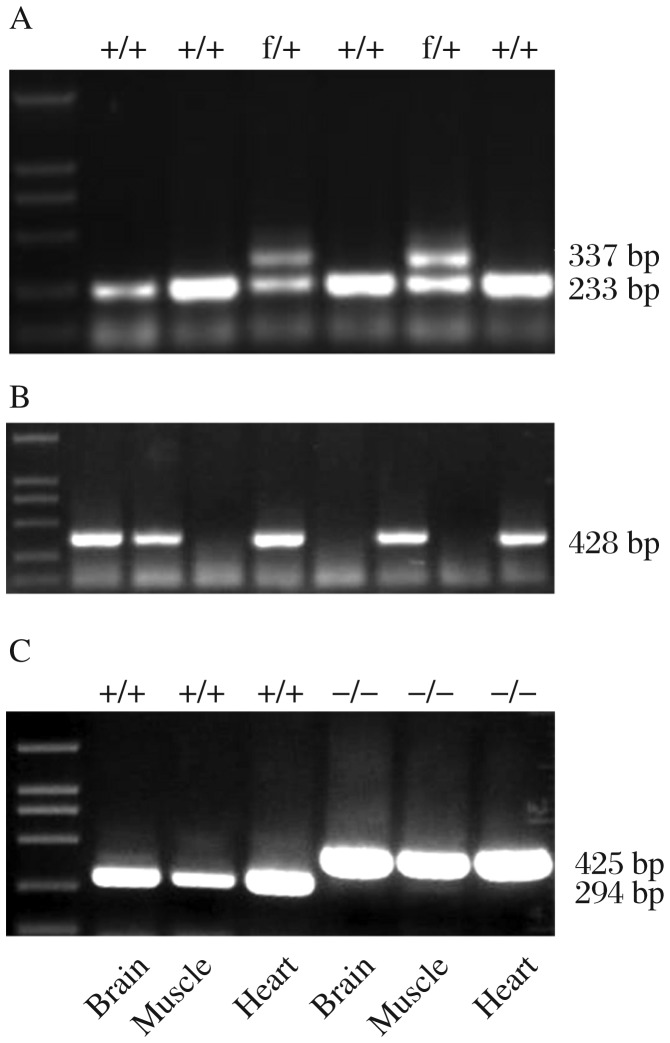
Genotyping by PCR assays. A: Genotyping the *PRL-3* flox allele by PCR. The PCR products are 233 bp (wild type) and 337 bp (PRL-3^flox^ alleles), respectively. B: Genotyping the PRL-3^Δexon4^ allele by PCR. The PCR products are 428 bp, representing the PRL-3^Δexon4^ allele. C: Reverse transcription PCR (RT-PCR) results. cDNA samples synthesized from RNA were extracted from the heart, muscle and brain tissues of the *PRL-3*^−/−^ mice and *PRL-3*^+/+^ mice. The PCR products are 425 bp (wildtype) and 294 bp (*PRL-3*^−/−^ alleles), respectively.

Targeted clones were further confirmed by Southern blot with 5′-and 3′-probes (***Fig. 2***). Introduction of an additional *Eco*RI restriction enzyme site was used to differentiate wildtype (22 kb) versus *PRL-3*-flox mutated allele (12.7 kb, 8.5 kb) (***Fig. 2A, 2B***). After confirmation by Southern blots, positive clones were then used to produce chimera by c57BL/6J blastocyst injection. Twenty-one chimeras (10 females and 11 males) were obtained. After crossing with c57BL/6J mice, 4 out of 10 female chimeric mice and 5 out of 11 male chimeric mice were identified to be capable of germline transmission. The pups of chimeric mice were also genotyped by Southern blot to check whether they contained the loxP mutation. To test Cre-mediated recombination of the *PRL-3*floxed allele, we crossed *PRL-3*^floxed^ mice with EIIA-Cre transgenic mice (FVB/N-TgN(EIIa-Cre)C5379Lmgd, the Jackson Laboratory). The pups (PRL-3^+/−^ mice) that had undergone Cre-mediated recombination were identified by PCR (***Fig. 3A***). Then, PRL-3^+/−^ mice were back-crossed to c57BL/6J background. PRL-3^−/−^ mice were obtained by inter-crossing of heterozygous mice (***Fig. 3B***).

**Fig. 4:**
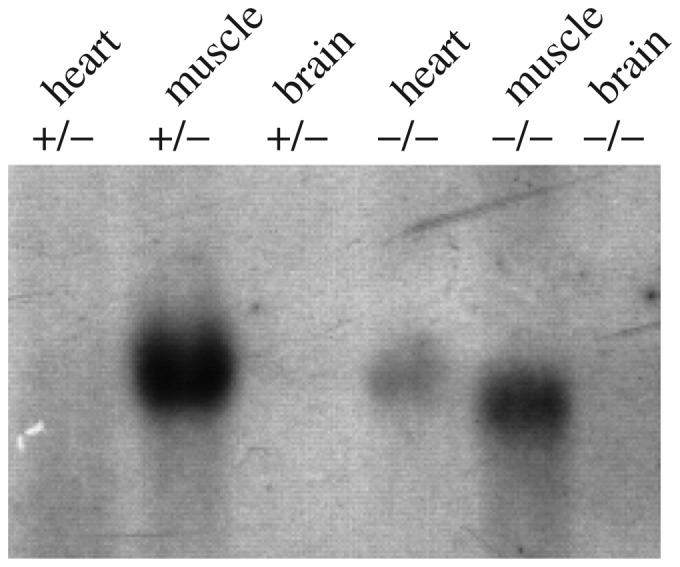
Northern blot analysis. Total RNA of the heart, brain and muscle were extracted from wildtype and PRL-3 heterozygous mice, respectively.

RT-PCR analysis of PRL-3 expression in the heart, muscle and brain tissues of wildtype mice and *prl*-3^−/−^ mice were performed simultaneously. The PCR products from the tissues of *PRL*-3^−/−^ mice were 294 bp while the PCR products from the wildtype mice were 425 bp (***Fig. 3C***). Northern blot analysis of prl-3 expression in the heart, muscle and brain tissues of wildtype mice and *prl*-3^+/−^ mice were performed simultaneously (***Fig. 4***).

**Fig. 5:**
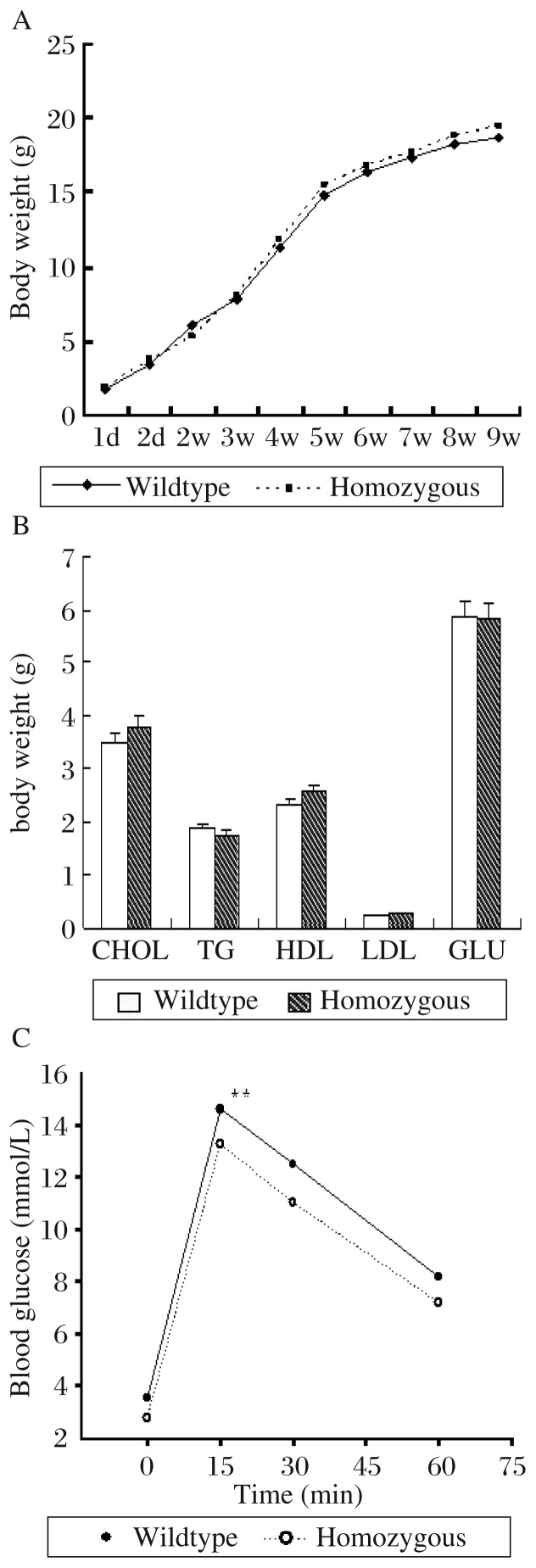
Phenotypic analysis of the *PRL-3*^−/−^ mice and *PRL-3*^+/+^ mice. A: Growth curve of the *PRL-3*^−/−^ mice and *PRL-3*^+/+^ mice. Each group had 9 mice and their body weight was measured at 1, 2, 3, 4, 5, 6, 7, 8, and 9 weeks. There was no significant difference between the PRL-3^−/−^ mice and PRL-3^+/+^ mice (*P* > 0.05). B: Analysis of blood chemistry indicators in plasma from PRL-3^−/−^ and PRL-3^+/+^ mice. We detected cholesterol (CHOL), triglycereide (TG), high density lipoproteins (HDL), low density lipoproteins (LDL) and glucose (GLU). There was no significant difference in blood chemistry indications of homozygous type and wildtype (*P* > 0.05). C: Intraperitoneal glucose tolerance test (IPGTT). Twelve-week-old *PRL-3*^−/−^ and *PRL-3*^+/+^ mice were selected, respectively, and each group had 8 mice. Measurement of glucose level was performed after 16 h fasting. Mice were intraperitoneal injected with D-α glucose (0.15 g/mL, diluted in MillQ) at a dose of 1.5 mg/kg. Then, the value of blood glucose was measured at 0, 15, 30, 45 and 60 min by using Blood Glucose Test Strips. The glucose level of the *PRL-3*^−/−^ mice was significantly lower than that of the *PRL-3*^+/+^ mice after 16 h fasting.

The growth curve of *PRL-3*^−/−^ mice and *PRL-3*^+/+^ mice exhibited no significant difference (*P* > 0.05) (***Fig. 5A***). Blood chemistry indicators of *PRL-3*^−/−^ mice and *PRL-3*^+/+^ mice were measured. We detected cholesterol, triglycereide, high density lipoproteins, LDL and glucose. There was no significant difference in blood chemistry indicators in the homozygous type and wide type (*P* > 0.05, ***Fig. 5B***). The IPGTT results suggested that the glucose level of PRL-3^−/−^ mice was significantly lower than of *PRL-3*^+/+^ mice after 16 h fasting (***Fig. 5C***).

## DISCUSSION

PRL-3 has been shown to play an important role in promoting cell migration and tumor metastasis. It has been reported that PRL-3 exerts its functions by modulating the Rho family GTPase, activating Src, and regulating the PI3K-Akt pathway in a context-dependent manner[20]. In this study, we applied a rapid and efficient method for constructing conditional knockout alleles for *PRL-3* that is based on *E. coli* recombineering rather than restriction enzymes and DNA ligases.

To test whether the neo gene and exon 4 in the *PRL-3* floxed allele had been deleted, the heterozygous(*PRL-3* floxed) mice with Cre recombinase were crossed with the EIIa-Cre transgenic mice which express Cre recombinase in the germline. The offspring *PRL-3*^+/−^ mice were identified by PCR products. Moreover, to further determine the accuracy of PRL-3 recombination in mice, we generated homozygous PRL-3 null mice. To demonstrate correct *PRL-3* knockout mice, we detected *PRL-3* mRNA expression in the heart, muscle and brain tissues isolated from wildtype mice and *PRL-3*^−/−^ mice simultaneously. Additionally, the sequencing results and Northern blot analysis demonstrated successful knockout in mRNA level (data not shown). However, Western blotting examination of protein expression in the tissues extracted from wildtype and *PRL-3*^−/−^ mice separately could not be obtained. Only in the metastasis tissues or tumors extracted from the wildtype mice, high expression level of PRL-3 protein could have been proved. Previous reports suggested that PRL-3 is the only gene consistently overexpressed in 100% of 18 colorectal cancer liver metastases, which indicates that PRL-3 plays an important role in tumor metastasis[4],[21].

Further tests were performed to screen the phenotype of the *PRL-3* knockout mice. We established growth curve for *PRL-3*^−/−^ mice and *PRL-3*^+/+^ mice and found no significant difference in their body weights. Blood chemistry indicators of *PRL-3*^−/−^ mice and *PRL-3*^+/+^ mice were also measured. Finally, we compared the levels of glucose and indicators of lipid metabolism related factors between the homozygous and wildtype mice, and found no significant difference. However, PRL-3^−/−^ mice exhibited abnormal response to IPGTT, which indicated that the mice might be used for further glucose metabolism related investigations. It has been previously reported that PRL-3 has some homology with PTEN in the phosphatase active site and flanking regions, and may act as a lipid phosphatase at the cytoplasmic face of the plasma membrane[21],[22].

In conclusion, we successfully generated the conditional knockout alleles for PRL-3, allowing Cre-mediated inactivation of PRL-3 during mouse development and in specific adult tissues. Moreover, our conditional *PRL-3* knockout mice will be a valuable tool for uncovering the role of PRL-3 and its substrates in tumor metastasis that may be new potential therapeutic targets for cancer treatment.

## References

[1] Ostman A, Hellberg C, Böhmer FD (2006). Protein-tyrosine phosphatases and cancer. Nat Rev Cancer.

[2] Zeng Q, Hong W, Tan YH (1998). Mouse PRL-2 and PRL-3, two potentially prenylated protein tyrosine phosphatases homologous to PRL-1. Biochem Biophys Res Commun.

[3] Zeng Q, Si X, Horstmann H, Xu Y, Hong W, Pallen CJ (2000). Prenylation-dependent association of protein-tyrosine phosphatases PRL-1, -2, and -3 with the plasma membrane and the early endosome. J Biol Chem.

[4] Saha S, Bardelli A, Buckhaults P, Velculescu VE, Rago C, St Croix B (2001). A phosphatase associated with metastasis of colorectal cancer. Science.

[5] Peng L, Ning J, Meng L, Shou C (2004). The association of the expression level of protein tyrosine phosphatase PRL-3 protein with liver metastasis and prognosis of patients with colorectal cancer. J Cancer Res Clin Oncol.

[6] Zhao GP, Zhou ZG, Lei WZ, Yu YY, Zheng XL, Gao HK (2005). Expression of phosphatase of regenerating liver-3 mRNA and its clinical implications in human colorectal carcinoma. Zhonghua Wei Chang Wai Ke Za Zhi.

[7] Wu X, Zeng H, Zhang X, Zhao Y, Sha H, Ge X (2004). Phosphatase of regenerating liver-3 promotes motility and metastasis of mouse melanoma cells. Am J Pathol.

[8] Fagerli UM, Holt RU, Holien T, Vaatsveen TK, Zhan F, Egeberg KW (2008). Overexpression and involvement in migration by the metastasis-associated phosphatase PRL-3 in human myeloma cells. Blood.

[9] Yamashita S, Masuda Y, Matsumoto K, Okumura Y, Matsuzaki H, Kurizaki T (2007). Down-regulation of the human PRL-3 gene is associated with the metastasis of primary non-small cell lung cancer. Ann Thorac Cardiovasc Surg.

[10] Wang Y, Li ZF, He J, Li YL, Zhu GB, Zhang LH (2007). Expression of the human phosphatases of regenerating liver (PRLs) in colonic adenocarcinoma and its correlation with lymph node metastasis. Int J Colorectal Dis.

[11] Miskad UA, Semba S, Kato H, Matsukawa Y, Kodama Y, Mizuuchi E (2007). High PRL-3 expression in human gastric cancer is a marker of metastasis and grades of malignancies: an in situ hybridization study. Virchows Arch.

[12] Li Z, Zhan W, Wang Z, Zhu B, He Y, Peng J (2006). Inhibition of PRL-3 gene expression in gastric cancer cell line SGC7901 via microRNA suppressed reduces peritoneal metastasis. Biochem Biophys Res Commun.

[13] Wang H, Quah SY, Dong JM, Manser E, Tang JP, Zeng Q (2007). PRL-3 down-regulates PTEN expression and signals through PI3K to promote epithelial-mesenchymal transition. Cancer Res.

[14] Stephens BJ, Han H, Gokhale V, Von Hoff DD (2005). PRL phosphatases as potential molecular targets in cancer. Molecular cancer therapeutics.

[15] Bardelli A, Saha S, Sager JA, Romans KE, Xin B, Markowitz SD (2003). PRL-3 expression in metastatic cancers. Clin Cancer Res.

[16] Miskad UA, Semba S, Kato H, Yokozaki H (2004). Expression of PRL-3 phosphatase in human gastric carcinomas: close correlation with invasion and metastasis. Pathobiology.

[17] Liu P, Jenkins NA, Copeland NG (2003). A highly efficient recombineering-based method for generating conditional knockout mutations. Genome Res.

[18] Lee EC, Yu D, Martinez de Velasco J, Tessarollo L, Swing DA, Court DL (2001). A highly efficient Escherichia coli-based chromosome engineering system adapted for recombinogenic targeting and subcloning of BAC DNA. Genomics.

[19] Zhang YL, Tan XH, Xiao MF, Li H, Mao YQ, Yang X (2004). Establishment of liver specific glucokinase gene knockout mice: a new animal model for screening anti-diabetic drugs. Acta Pharmacol Sin.

[20] Peng L, Xing X, Li W, Qu L, Meng L, Lian S (2009). PRL-3 promotes the motility, invasion, and metastasis of LoVo colon cancer cells through PRL-3-integrin beta1-ERK1/2 and-MMP2 signaling. Mol Cancer.

[21] Zeng Q, Dong JM, Guo K, Li J, Tan HX, Koh V (2003). PRL-3 and PRL-1 promote cell migration, invasion, and metastasis. Cancer Res.

[22] Diamond RH, Cressman DE, Laz TM, Abrams CS, Taub R (1994). PRL-1, a unique nuclear protein tyrosine phosphatase, affects cell growth. Mol Cell Biol.

